# Potential impact of [^18^F]-FACBC PET in radiotherapy target definition of glioma

**DOI:** 10.1186/s13014-025-02752-2

**Published:** 2025-11-21

**Authors:** Benedikte Emilie Vindstad, Tora Skeidsvoll Solheim, Josefine Ståhl-Kornerup, Ole Skeidsvoll Solheim, Erik Magnus Berntsen, Lars Kjelsberg Pedersen, Anna Maria Karlberg, Live Eikenes

**Affiliations:** 1 Department of Circulation and Medical Imaging, Norwegian University of Science and Technology, Trondheim, Norway; 2 The Cancer Clinic, St. Olavs Hospital, Trondheim University Hospital, Trondheim, Norway; 3 Department of Clinical and Molecular Medicine, Norwegian University of Science and Technology, Trondheim, Norway; 4 Department of Radiotherapy, The Cancer Clinic, St Olavs Hospital, Trondheim University Hospital, Trondheim, Norway; 5 Department of Neurosurgery, St. Olavs Hospital, Trondheim University Hospital, Trondheim, Norway; 6 Department of Neuromedicine and Movement Science, Norwegian University of Science and Technology, Trondheim, Norway; 7 Department of Radiology and Nuclear Medicine, St. Olavs Hospital, Trondheim University Hospital, Trondheim, Norway; 8 Department of Neurosurgery, University Hospital of North Norway, Tromsø, Norway

**Keywords:** PET, Radiation oncology, Radiotherapy planning, Target volume delineation, Glioma

## Abstract

**Purpose:**

The aim of the study was to compare [^18^F]-FACBC positron emission tomography (PET)-based radiotherapy (RT) volumes to magnetic resonance (MR)-based volumes and investigate the potential impact of including [^18^F]-FACBC PET in RT treatment planning for gliomas.

**Methods:**

MR- and PET-defined gross tumor volumes (GTVs) were independently contoured on pre-operative [^18^F]-FACBC PET/MR images in 24 patients with primary or recurrent low- or high-grade glioma. MR GTVs were defined from regions of contrast-enhancement on T1-weighted (ce-T1) sequences. For non-enhancing tumors or non-enhancing tumor components, regions of FLAIR hyperintensity were also included. PET-based GTVs were delineated using a tumor-to-background ratio (TBR) threshold of 2. GTVs were expanded by an isotropic margin to form clinical target volumes (CTVs). Volumetric analysis was performed using size comparisons, Dice coefficients (DC), overlap coefficients (OC) and Hausdorff distances (HD).

**Results:**

PET GTVs were overall significantly smaller than MR GTVs, with median values of 14.8 ccm (6.5–26.7 ccm) and 28.5 ccm (17.2–62.7 ccm), respectively (*p* = 0.011). No significant volume difference was found between PET and MR volumes when MR volumes were based on ce-T1 only, but PET-based GTVs and CTVs were significantly smaller than MR-based GTVs and CTVs when the latter included FLAIR hyperintensity (*p* < 0.01). Similarity metrics showed a high degree of concordance between PET and MR volumes in cases where MR volumes were based on ce-T1 only, particularly for CTVs (DC: 0.88 (0.82–0.94), OC: 0.98 (0.96–0.99), HD: 0.86 cm (0.7–1.4 cm)). In comparison, for cases including FLAIR hyperintensity, MR CTVs had a high overlap with PET CTVs (OC: 0.99 (0.96–0.99)), but otherwise significantly lower degree of concordance (DC: 0.66 (0.46–0.81), *p* < 0.01, HD: 2.65 cm (2.22–3.85 cm), *p* < 0.001).

**Conclusion:**

[^18^F]-FACBC PET underestimates tumor extension compared to FLAIR in contrast negative tumors and tumors containing non-enhancing components, indicating reduced sensitivity to low-grade tumor tissue and microscopic tumor infiltration. Inclusion of [^18^F]-FACBC PET as supplement to MR in RT planning for glioma could however help identify regions of highly malignant tumor, to facilitate target volume reduction and dose escalation strategies.

## Introduction

Gliomas are the most common primary malignant brain cancer in adults, and are associated with high rates of recurrence and substantial morbidity and mortality [1]. Radiotherapy (RT) in combination with chemotherapy has an established role in glioma treatment, both following surgery and in the event of recurrence [2]. Accurate target delineation is important for improving the effectiveness of RT while reducing dose to normal brain tissue.

In RT planning of gliomas, gross tumor volume (GTV) is typically defined based on magnetic resonance (MR) images. To account for tumor infiltration into surrounding tissue, GTVs are expanded by a margin (1–2 cm) to obtain the clinical target volume (CTV) [3, 4]. Grade 2 and grade 3 tumors are often non-enhancing, and GTV delineation is based on T2 fluid-attenuated inversion-recovery (FLAIR) signal abnormalities for these tumors. For grade 4 tumors GTVs are defined primarily based on contrast-enhanced regions on T1-weighted MR images (ce-T1), which indicates tumor tissue through disruption of the blood-brain barrier (BBB). Although they typically present with some contrast enhancement, grade 4 tumors also often contain non-enhancing regions [5, 6]. In these cases, GTV delineation is supplemented by FLAIR images. However, FLAIR has some limitations when it comes to differentiation of tumor tissue from edema, inflammation, postoperative ischemic changes or gliosis, and identification of microscopic tumor infiltration. Furthermore, while smaller CTV margins are sometimes used for FLAIR-based GTVs in non-enhancing lower-grade gliomas, there is currently no consensus on whether margins can be similarly reduced for non-enhancing tumor regions in glioblastomas. The inclusion of FLAIR to delineate GTVs therefore often translates to large treatment volumes, which raises concerns regarding increased toxicity in the normal brain. To limit RT-induced neurocognitive toxicity, recent guidelines recommend reduced CTVs through more conservative margins and exclusion of non-tumor FLAIR hyperintensity if possible, but delineation of GTV in non-enhancing tumor regions remains a challenge [4].

Amino acid (AA) positron emission tomography (PET) can provide additional information about the metabolic activity of gliomas and is recommended as a supplement to MR in RT target delineation [7, 8]. AA tracer *anti*-1-amino-3-[^18^F]-fluorocyclobutane-1-carboxylic acid ([^18^F]-FACBC) has previously shown uptake in most glioma types [9], also in contrast negative tumors, with low uptake in normal brain tissue and high tumor-to-background (TBR) values compared to other AA tracers [10, 11]. Recently, histomolecular validation of [^18^F]-FACBC PET showed high accuracy for identification of glioma tissue, particularly for high-grade tumor tissue [12]. This indicates [^18^F]-FACBC PET as a potential supplement to MR in RT for glioma, to help identify regions of high malignancy also in the absence of contrast enhancement. This metabolic tumor volume could be used in RT of glioma to better delineate GTVs or identify targets for dose escalation (DE) strategies. However, it is unknown whether the size and location of [^18^F]-FACBC PET uptake significantly differs from tumor volumes defined from ce-T1 or FLAIR in the context of determining RT GTVs. The aim of this study was to determine to what extent MRI and [^18^F]-FACBC PET based GTVs and CTVs differ and to investigate the potential impact of including [^18^F]-FACBC PET in treatment planning for gliomas. The study was performed retrospectively, and PET based GTVs and CTVs were not used in the RT planning for the included patients.

## Methods

### Patients

Adult patients with suspected primary or recurrent diffuse glioma (*n* = 48) were recruited for pre-operative [^18^F]-FACBC PET/MR from the Department of Neurosurgery, St. Olavs Hospital, Trondheim University Hospital, Trondheim, and from the Department of Neurosurgery, University Hospital of North Norway, Tromsø, between May 2016 and June 2021. Exclusion criteria were pregnancy, breastfeeding, pacemakers or defibrillators not compatible with 3 Tesla (T) MRI, no ability to obtain informed consent (e.g., due to severe dysphasia or cognitive deficits), weight > 120 kg and Karnofsky performance status < 70. Six patients were excluded due to withdrawn consent (*n* = 1) or problems with tracer delivery (*n* = 5), and the remaining 42 patients underwent pre-operative [^18^F]-FACBC PET/MR. Further exclusion criteria for the current study were grade 1 gliomas (*n* = 2), uncertain diagnosis/subtype (*n* = 5), and PET negative gliomas (*n* = 10), leaving 25 patients for data analysis.

All participants gave written informed consent. The study was approved by the Regional Ethics Committee (REK South East Norway, reference numbers: 2016/279 and 2018/2243).

### Histomolecular diagnosis

Histomolecular diagnosis was determined according to the 2021 World Health Organization (WHO) classification of Central Nervous System (CNS) tumors. Biopsy samples from patients included prior to the introduction of the WHO 2021 classification were reclassified according to the 2021 criteria. Of the included patients, 15 were diagnosed with IDH wildtype glioblastoma (grade 4), five with IDH-mutant astrocytoma (grade 3: *n* = 3, grade 4: *n* = 2), and five with IDH-mutant oligodendroglioma (grade 2: *n* = 2, grade 3: *n* = 3).

### [^18^F]-FACBC PET/MR imaging

Two identical hybrid PET/MR systems (Siemens Biograph mMR, software version Syngo MR VE11P, Erlangen, Germany) were used for simultaneous PET and MR acquisitions. Patients were injected with [^18^F]-FACBC (3 ± 0.2 MBq/kg) on the scanner examination table at the start of the PET and MRI imaging, and list-mode PET was acquired 0 to 45 min post injection (p.i.).

MRI sequences were acquired according to current consensus recommendations on standardized brain tumor imaging protocols [13, 14]. These included pre– and post–contrast-enhanced 3D T1 magnetization prepared rapid gradient echo imaging (MPRAGE), 3D FLAIR and T2, as well as an ultrashort echo time (UTE) sequence for PET attenuation correction (AC) purposes.

Static PET images (30–45 min p.i.) were reconstructed with iterative reconstruction (3D OSEM algorithm, 3 iterations, 21 subsets, 344 × 344 matrix, 4-mm Gaussian filter) with point spread function correction, decay correction, scatter correction, and AC. AC was based on the UTE sequence and deep learning method DeepUTE developed by Ladefoged et al. [15, 16]. For one patient, the examination was interrupted at 29 min p.i., and reconstruction at 14–29 min p.i. was used instead.

### Target volume delineation

For the purposes of this study, separate MR and PET RT target volumes were retrospectively generated based on pre-operative [^18^F]-FACBC PET/MR images. Thus, both MR and PET target volumes in this study were theoretical and were not used for RT planning for the included patients. To ensure a robust basis of comparison, MR and PET target volumes were generated independently of each other following the most recent guidelines from the European Organisation for Research and Treatment of Cancer (EORTC) [4]. Any pre-existing clinical RT volumes for these patients were not assessed during delineation.

For all patients, ce-T1 and FLAIR tumor volumes were first defined manually by a neuroradiologist and a physicist together. MR GTVs and CTVs were subsequently generated based on the ce-T1 and/or FLAIR volumes guided by a neurooncologist. For 9 patients, GTVs were based on ce-T1 volumes only, as no non-enhancing tumor regions were suspected from the MR images. Three patients presented with no contrast enhancement (one of which did not have ce-T1 images available), and in these, MR GTVs were based on hyperintensity in FLAIR images only. The remaining 13 patients had partial contrast enhancement, and for these, FLAIR hyperintensity corresponding to suspected tumor were included in the GTV in addition to the ce-T1 volume. FLAIR signal determined to represent edema and other non-tumor abnormalities were excluded from GTVs. In recurrent gliomas, the surgical cavity was included in the GTV if present.

The PET based GTV was defined by applying a TBR ≥ 2 threshold for [^18^F]-FACBC uptake. The threshold was based on histopathological validation of [^18^F]-FACBC [12], where TBR ≥ 2 was found to be optimal for identification of high-grade glioma tissue based on image-localized biopsies. As no threshold for low-grade tissue has been determined, the same threshold was applied to both high-and low-grade gliomas in this study. PET GTVs were restricted to brain tissue.

Both PET- and MR based CTVs were defined by adding a 1 cm (grade 2 gliomas) or 1.5 cm (grade 3–4 gliomas) margin to their respective GTVs, according to the latest recommendations for CTV definition in gliomas [3, 4]. The CTVs were restricted to brain tissue and cut off with a 0.5 cm margin into the cerebellum and/or opposite hemisphere, except in cases where the GTV also extended across both hemispheres. The brainstem was excluded from the CTVs.

### Statistical analysis

To compare differences in volume sizes and similarity metrics, Wilcoxon signed-rank tests were used. P-values of main effects were adjusted for multiple comparisons using Benjamini & Hochberg’s correction for false discovery rate [17], with significance level set to *p* < 0.05. The effect size *r* was calculated and classified as 0.1–0.3 (small effect), 0.3–0.5 (moderate effect) and > 0.5 (large effect) [18]. All statistical calculations were performed in RStudio.

Spatial similarity between PET and MR GTVs and CTVs were evaluated by calculating the Dice similarity coefficient (DC), the overlap coefficient (OC) and the Hausdorff distance (HD). The DC [19] was calculated as:

$$\:DC\left(A,B\right)=\frac{2\left(A\bigcap\:B\right)}{\left(A+B\right)}$$

Where A was the PET GTV or CTV, and B was the MR GTV or CTV. Values close to 0 indicate low spatial correlation, both in position and size, while values close to 1 indicate high similarity. A DC of 1 would describe two volumes of equal size, shape, and placement.

The overlap coefficient was calculated as:

$$\:OC(A,B)=\frac{A\bigcap\:B}{\text{m}\text{i}\text{n}(A,B)}$$

Values close to 0 indicate low degree of overlap, while values close to 1 indicate that one volume is mostly contained within the other.

The HD was calculated as the greatest of all distances from a surface point of either volume to the closest surface point in the other volume [20]:

$$\:HD\left(A,B\right)=\text{max}\left(\text{h}\left(\text{A},\text{B}\right),\text{h}\left(\text{B},\text{A}\right)\right)\:$$

$$\:h\left(A,B\right)=\underset{a\in{A}}{\text{max}}\:\underset{b{\in}B}{\text{min}}\left\| {a-b} \right\|$$

The HD shows the difference in the contours at their most mismatched point. A small HD indicates that the volume boundaries are well matched at all points. A large HD indicates a large mismatch between volume boundaries in at least one area.

## Results

Tumor characteristics and PET and MR GTVs and CTVs for each patient are shown in Table 1.

**Table 1:** Tumor characteristics and PET and MR GTVs and CTVs (ccm)

Grade	Diagnosis	Primary/ recurrent	Ce-T1 only MR volumes	PET GTV	MR GTV	PET CTV	MR CTV
2	Oligodendroglioma	Primary	No	57.9	166.3	242.5	334.6
2	Oligodendroglioma	Recurrent	No	0.3	29.9	12.1	94.3
3	Astrocytoma	Primary	No	0.04	151.3	21.3	385.8
3	Astrocytoma	Primary	No	13.3	60.0	111.4	190.7
3	Astrocytoma	Recurrent	Yes	27.5	13.2	125.4	114.9
3	Oligodendroglioma	Primary	No	1.8	25.4	35.2	127.0
3	Oligodendroglioma	Recurrent	No	1.6	17.7	53.2	99.9
3	Oligodendroglioma	Recurrent	No	25.3	69.0	169.6	415.2
4	Astrocytoma	Primary	No	7.4	76.8	97.9	404.8
4	Astrocytoma	Recurrent	No	53.6	123.0	274.8	405.8
4	Glioblastoma	Primary	Yes	5.0	1.4	52.9	40.2
4	Glioblastoma	Primary	Yes	11.9	9.1	74.9	78.6
4	Glioblastoma	Primary	No	13.5	15.9	93.6	118.4
4	Glioblastoma	Primary	No	16.1	56.5	89.3	187.1
4	Glioblastoma	Primary	Yes	63.1	79.9	281.0	260.0
4	Glioblastoma	Primary	Yes	9.1	1.2	83.3	45.1
4	Glioblastoma	Primary	No	21.3	25.9	101.9	153.8
4	Glioblastoma	Primary	No*	16.9	60.5	120.8	250.7
4	Glioblastoma	Primary	No	1.4	40.4	44.5	143.6
4	Glioblastoma	Primary	Yes	7.0	11.5	68.7	105.9
4	Glioblastoma	Primary	No	29.9	19.4	136.6	134.0
4	Glioblastoma	Primary	Yes	39.2	22.3	214.5	166.7
4	Glioblastoma	Recurrent	No	43.9	52.3	241.4	267.2
4	Glioblastoma	Recurrent	Yes	18.6	27.1	106.6	136.1
4	Glioblastoma	Recurrent	Yes	26.4	18.5	104.0	100.4
Median (IQR)				16.1 (7.0–27.5)	27.1 (17.7–60.5)	101.1 (68.7–136.6)	143.6 (105.9–260.0)

*contrast MRI not performed

GTV, gross tumor volume; CTV, clinical target volume, IQR: interquartile range

**Fig. 1:**
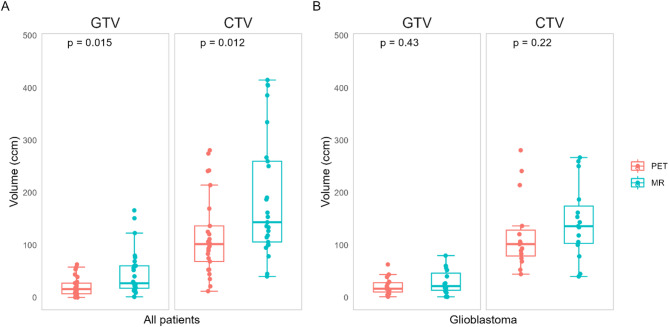
PET- and MR-based GTV and CTV volumes (ccm) for all patients, and for glioblastoma patients only. PET volumes were significantly smaller than MR volumes for both GTV (*p* = 0.015) and CTV (*p* = 0.012) in all patients. There was no significant difference in volumes for glioblastomas alone. GTV, gross tumor volume; CTV, clinical target volume

Median MR GTV and PET GTV values were 27.1 ccm (interquartile range (IQR) 17.7–60.5 ccm) and 16.1 ccm (IQR 7.0–27.5 ccm), respectively, while median MR CTV and PET CTV values were 143.6 ccm (IQR 105.9–260.0 ccm) and 101.1 ccm (IQR 68.7–136.6 ccm), respectively. Tumor volumes for all tumor types and for glioblastomas only are presented in Fig. 1. Statistical volume differences for astrocytomas and oligodendrogliomas were not considered due to small sample sizes. Overall, both PET GTVs and CTVs were significantly smaller than their MR based counterparts (GTV: *p* = 0.015, CTV: *p* = 0.012). For the glioblastomas only, PET GTVs/CTVs were smaller than MR GTVs/CTVs for 9/15 cases, but no significant volume difference was found (GTV: *p* = 0.43, CTV: *p* = 0.22).

**Fig. 2:**
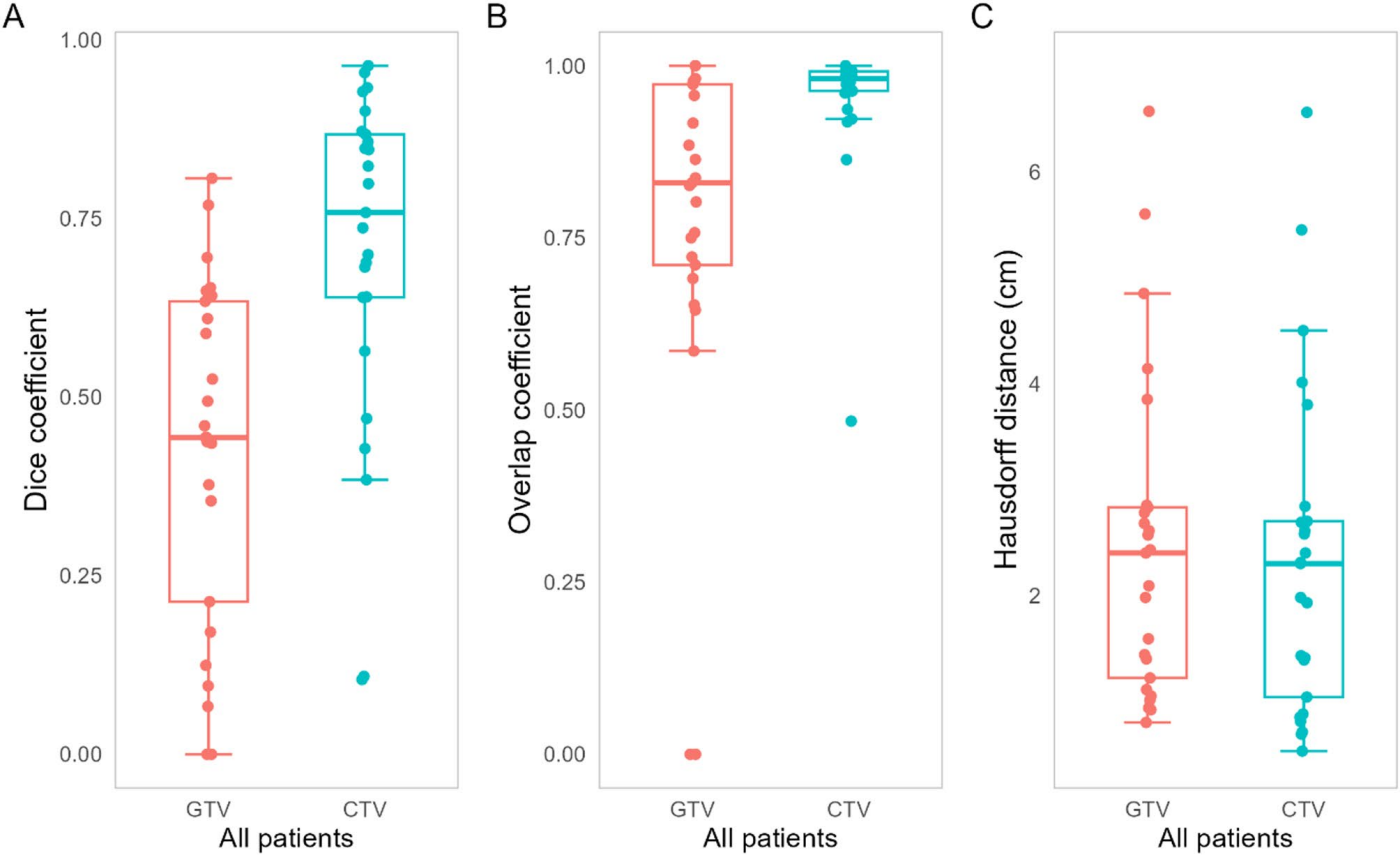
Similarity metrics for comparison of PET- and MR-based GTV and CTV volumes for all patients. GTV, gross tumor volume; CTV, clinical target volume

Similarity metrics for all patients are presented in Fig. 2. When comparing GTVs, the median Dice coefficient was 0.44 (IQR 0.21–0.63), the mean overlap coefficient was 0.83 (IQR 0.71–0.97), and the mean Hausdorff distance was 2.40 cm (IQR 1.22–2.83). For CTVs, the mean Dice coefficient was 0.76 (IQR 0.64–0.87), the mean overlap coefficient was 0.98 (IQR 0.96–0.99), and the mean Hausdorff distance was 2.30 cm (IQR 1.04–2.70).

**Fig. 3:**
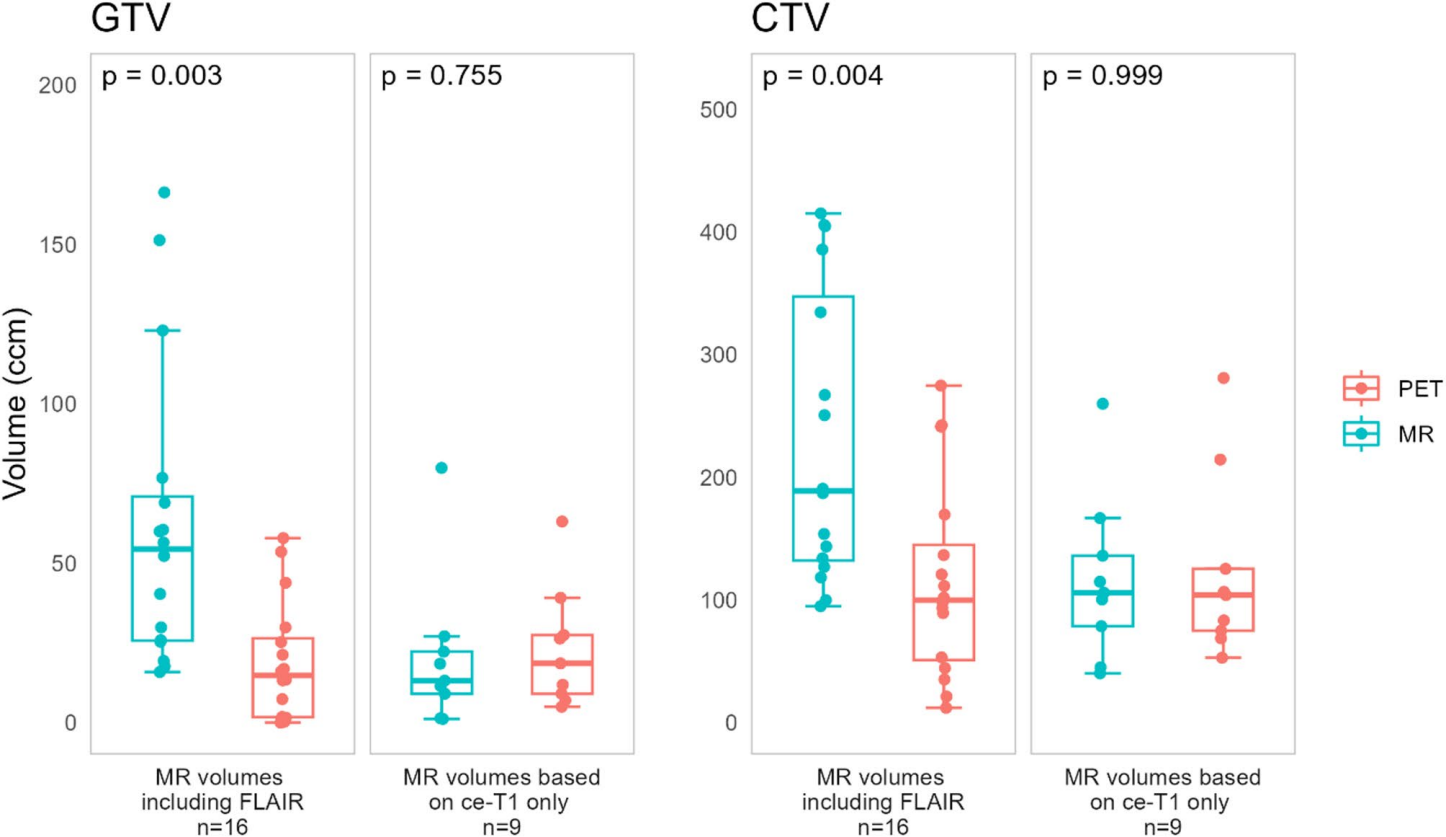
PET- and MR-based GTV and CTV volume (ccm), for MR volumes including FLAIR and based on contrast enhancement only. GTV, gross tumor volume; CTV, clinical target volume

Figure 3 shows the differences in PET and MR-based volumes for cases where MR volumes were based on ce-T1 images only, and for cases where MR volumes were based on FLAIR hyperintensity only or in addition to contrast enhancement. MR volumes including FLAIR hyperintensity were found to be significantly larger than the corresponding PET volumes (GTVs: *p* < 0.001, effect size 0.60 (large), CTVs: *p* < 0.01, effect size 0.53 (large)). No significant difference between PET and MR volumes was found for cases where the MR volumes were based on contrast enhancement only (GTVs: *p* = 0.755, CTVs: *p* = 0.999).

Similarity metrics for PET GTVs vs. MR GTVs, PET CTVs vs. MR CTVs, for ce-T1 only MR volumes and MR volumes including FLAIR hyperintensity, are presented in Table 2.

**Table 2:** DC, OC, and HD for PET GTVs vs. MR GTVs, PET CTVs vs. MR CTVs, for MR volumes including FLAIR hyperintensities and MR volumes based on ce-T1 only

		DC	OC	HD
MR volumes including FLAIR	**PET GTV vs. MR GTV** Median (IQR)	0.41 (0.12 - 0.52)	0.83 (0.72 - 0.96)	2.73 (2.42 – 3.92)
	**PET CTV vs. MR CTV** Median (IQR)	0.66 (0.46 - 0.81)	0.98 (0.95 - 0.99)	2.65 (2.22 - 3.85)
MR volumes based on ce-T1 only	**PET GTV vs. MR GTV** Median (IQR)	0.61 (0.44 - 0.64)	0.88 (0.69- 0.98)	1.05 (0.94 – 1.40)
	**PET CTV vs. MR CTV** Median (IQR)	0.87 (0.85 - 0.93)	0.98 (0.96 - 0.99)	0.85 (0.71 – 1.40)

DC, Dice coefficient; OC, overlap coefficient; HD, Hausdorff distance; GTV, gross tumor volume; CTV, clinical target volume; IQR, interquartile range

**Fig. 4:**
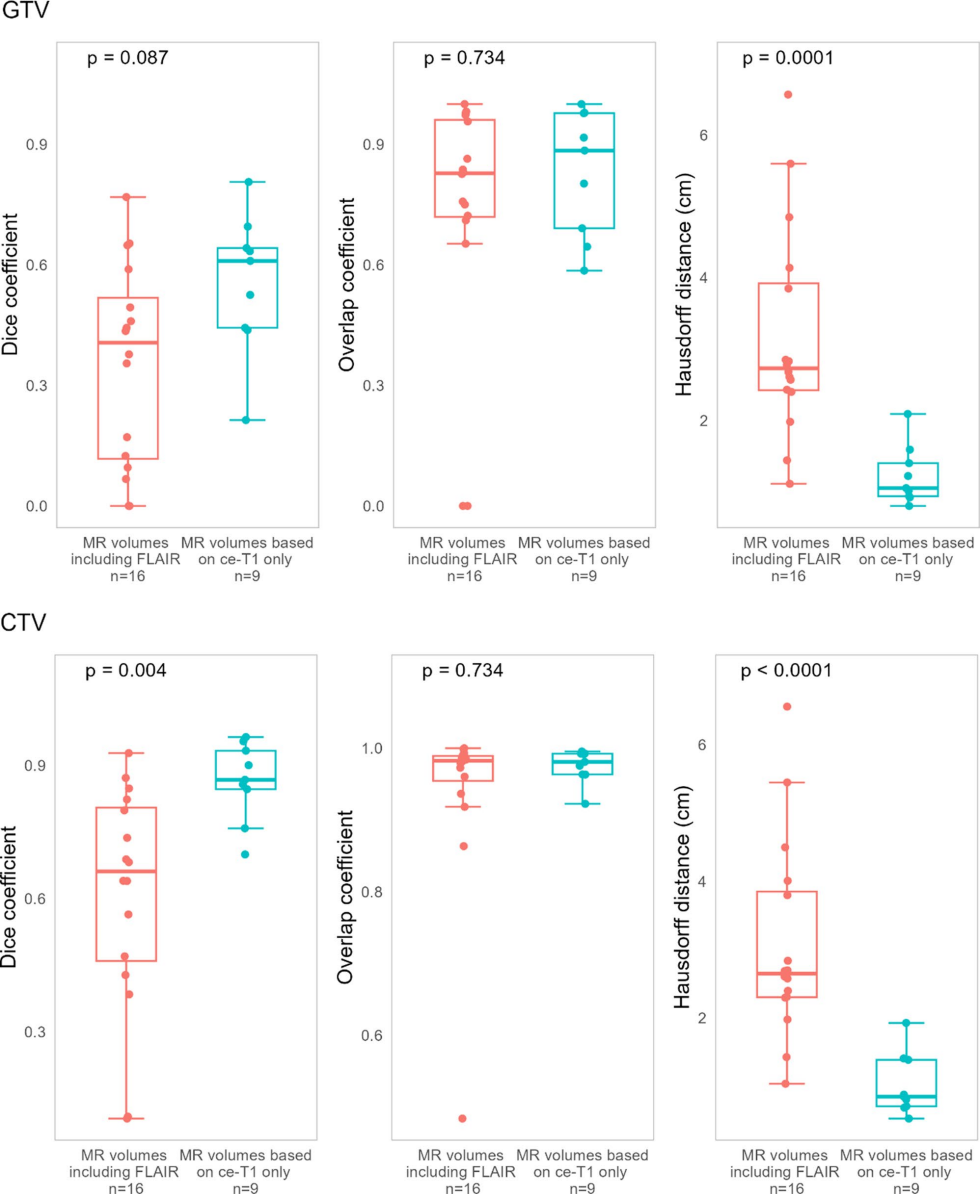
Similarity metrics for comparison of PET- and MR-based GTVs and CTVs, for cases where MR volumes were based on ce-T1 only and for cases where MR volumes included FLAIR hyperintensity. GTV, gross tumor volume; CTV, clinical target volume

**Fig. 5:**
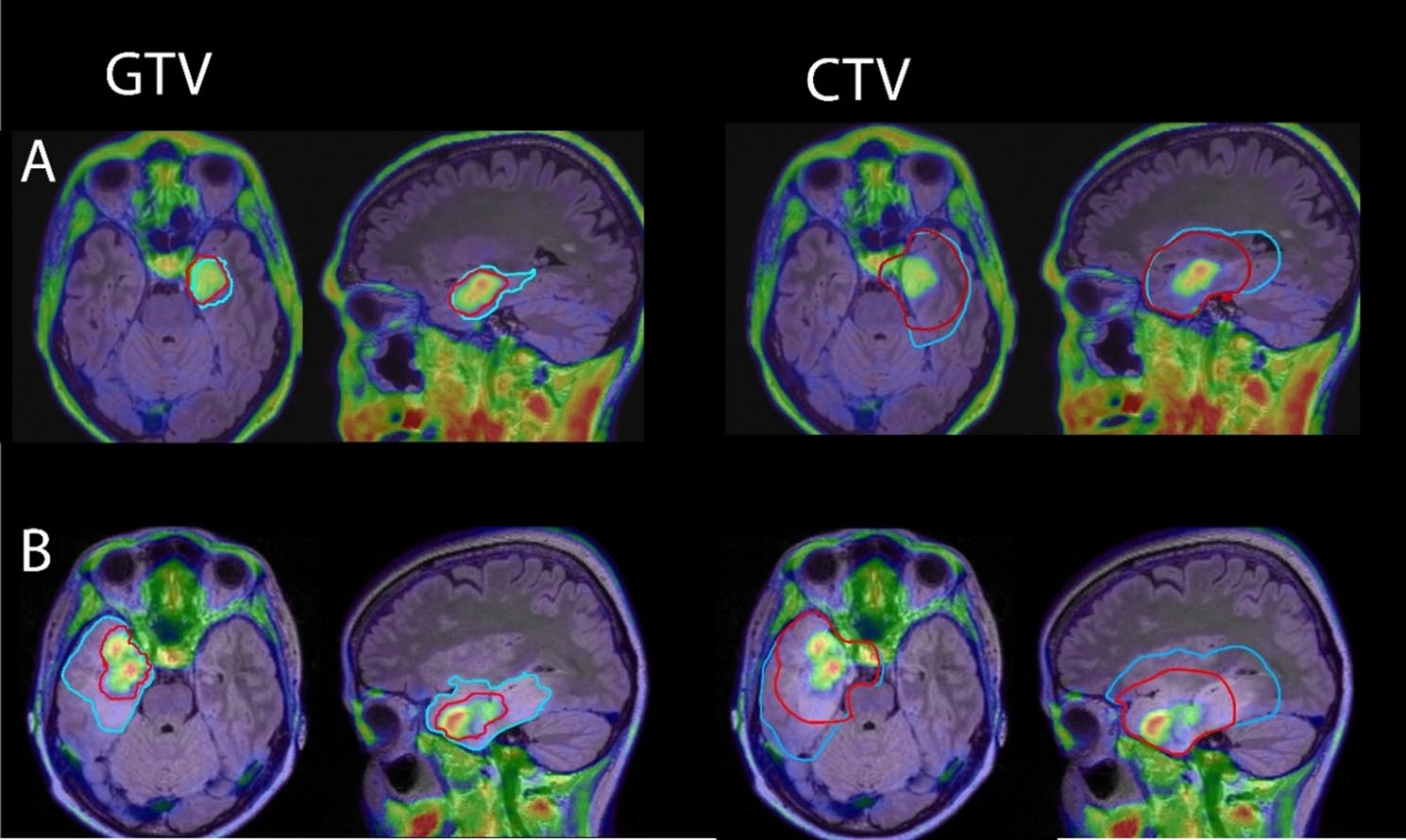
Examples of PET (red) and MR (blue) volumes in two patients with glioblastoma where MR GTVs included FLAIR hyperintensity due to lack of contrast uptake (**A**) or only partial contrast uptake (**B**). GTV, gross tumor volume; CTV, clinical target volume

**Fig. 6:**
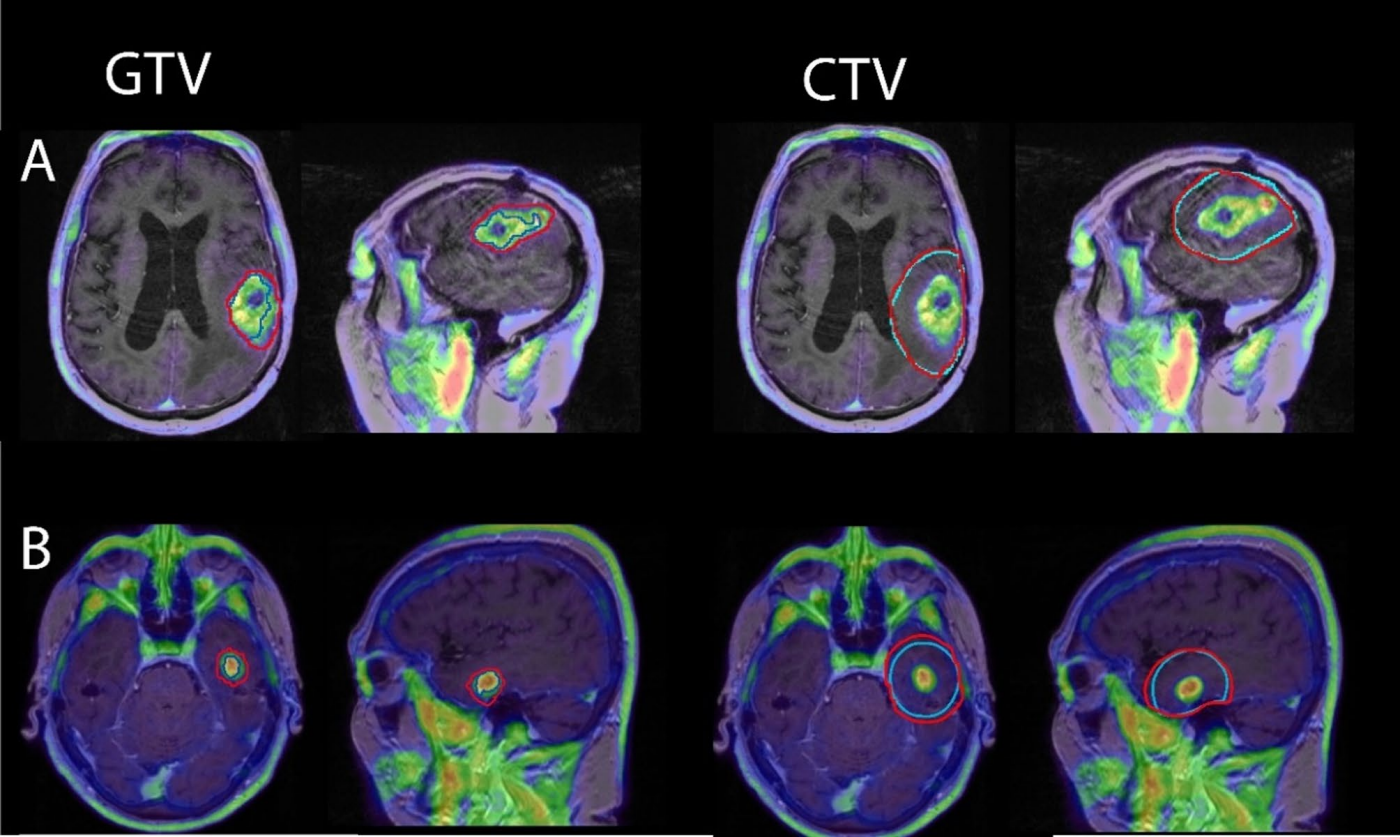
Examples of PET (red) and MR (blue) volumes in two patients with glioblastoma where MR GTVs were based on contrast uptake only. GTV, gross tumor volume; CTV, clinical target volume

Differences in similarity metrics between the two are shown in Fig. 4. PET and MR CTVs had significantly increased Dice values for ce-T1 only MR volumes (*p* = 0.004, effect size 0.60 (large)). PET and MR volumes had significantly decreased Hausdorff distances for both GTVs and CTVs for ce-T1 only MR volumes (GTVs: *p* = 0.0001, effect size 0.74 (large), CTVs: *p* < 0.0001, effect size 0.77 (large)). Examples of PET and MR volumes in patients where MR volumes included FLAIR hyperintensity are shown in Fig. 5, and examples of PET and MR volumes where MR volumes were based on ce-T1 images only are shown in Fig. 6.

## Discussion

To our knowledge, this is the first study to compare [^18^F]-FACBC PET and MR volumes in the context of RT planning for glioma. PET volumes were found to be significantly smaller than MR volumes for contrast negative tumors or tumors containing non-enhancing components, where MR GTVs included FLAIR hyperintensity in addition to the ce-T1 volume. For most of these cases, [^18^F]-FACBC volumes were contained within the MR volumes as shown by large overlap coefficients, while Dice coefficients and Hausdorff distances indicated a low degree of conformity between both GTVs and CTVs in this group, suggesting that [^18^F]-FACBC PET volumes were substantially different from the FLAIR volumes. In contrast-enhancing tumors without suspected non-enhancing components, [^18^F]-FACBC PET volumes were similar in size to the ce-T1-based volumes. Similarity metrics indicated some discordance between GTVs, but a high degree of conformity between CTVs, suggesting an overall large agreement between [^18^F]-FACBC PET uptake and contrast enhancement.

Accurate definition of non-enhancing tumor tissue is a topical challenge in RT treatment for glioma, and FLAIR hyperintensity plays an important role in RT target definition. While particularly relevant for grade 2 and 3 gliomas, this also applies for grade 4 gliomas, as they are sometimes contrast negative or present with non-enhancing tumor components in addition to contrast enhanced areas [3, 21]. Tissue sampling studies of glioblastoma have previously found high tumor burdens in these non-enhancing lesions for partially enhancing tumors [5, 6, 22], and current guidelines recommend that FLAIR hyperintensity should be included in treatment volumes for glioblastomas where these are suspected to represent non-enhancing tumor rather than edema [4]. In the current study, this applied to half (7/15) of the included glioblastomas.

AA PET is recommended for both lower-grade gliomas and glioblastomas to help differentiate non-enhancing tumor tissue from non-tumor FLAIR signal abnormalities, and to identify tumor infiltration not visible on MR [8]. However, our results illustrate key differences in tumor delineation in this context for [^18^F]-FACBC PET compared to [^11^C]-MET, [^18^F]-FET and [^18^F]-FDOPA. For these tracers, studies have shown significantly larger regions of PET uptake compared to MRI RT volumes based on both FLAIR and ce-T1, extending as far as 20–45 mm beyond contrast-enhanced volumes in some cases [23, 24]. Analysis of recurrence patterns showed that margins could be reduced while still covering recurrence sites in target volumes when including [^18^F]-FET uptake in GTVs [25], indicating good coverage of non-enhancing tumor spread. Similar results have been found for [^11^C]-MET and [^18^F]-FDOPA [26, 27]. Though RT volumes in these studies were based on postoperative PET/MR, studies using preoperative images also report similar findings [28–30], indicating that these tracers can consistently delineate metabolically active tumor beyond what is visible on MR images.

This was not seen for [^18^F]-FACBC in our study, as PET volumes largely agreed with ce-T1 only MR volumes and were significantly smaller than MR volumes for tumors including non-enhancing regions. This indicates that [^18^F]-FACBC PET may be less sensitive to microscopic tumor infiltration and low-malignancy non-enhancing tumor regions compared to FLAIR and other AA tracers, and likely underestimates true tumor extension in these cases. This is in line with previous results which found lower sensitivity for low-grade tumor tissue compared to high-grade tumor tissue for this tracer [12]. This difference in uptake patterns observed for the tracer compared to [^11^C]-MET, [^18^F]-FET and [^18^F]-FDOPA is likely due to differences in transport mechanisms, as [^18^F]-FACBC transport is mediated by the alanine-serine-cysteine transporter 2 (ASCT2) in addition to the l-type amino acid transporter 1 (LAT1) preferred by [^11^C]-MET, [^18^F]-FET and [^18^F]-FDOPA [31–33]. Both transporters are upregulated in glioma tissue, but ASCT2 is only expressed on the abluminal side of the BBB [34]. This could cause increased efflux of [^18^F]-FACBC across the barrier, leading to lower concentrations in the extracellular fluid in regions where the BBB has not been compromised, causing the low uptake seen in both normal tissue and low-malignancy regions. As the value of AA PET in informing RT treatment volumes depends largely on their ability to identify low-grade tumor spread and microscopic infiltration, our results indicate that [^18^F]-FACBC PET would be less effective than other AA tracers for this purpose.

Rather than delineating true tumor extension, [^18^F]-FACBC PET could be more suited for identification of highly malignant regions in non-enhancing tumor components, which could facilitate margin reduction or dose escalation strategies in RT of gliomas. Despite advances in treatment, gliomas continue to display high rates of central or in-field progression. Over 80% of grade 2 gliomas and grade 4 glioblastomas progress within the original RT field, also when treated with novel systemic agents [35, 36]. Also, with improved therapies extending survival for both grade 2/3 gliomas and glioblastomas, concerns about treatment-induced neurotoxicity have increased [37]. This has led to interest in reducing target volumes in RT for glioma. As recent studies using smaller CTV margins have reported comparable outcomes in terms of overall survival, progression-free survival, and recurrence patterns, newer guidelines and research support reduced margins for both lower-grade gliomas and glioblastomas, to help minimize toxicity without compromising tumor control [38–41]. However, there is currently no consensus regarding the margin that should be applied to non-enhancing FLAIR volume in glioblastomas while adequately accounting for tumor infiltration, with recommendations ranging from 0 to 1.5 cm. While the current study indicates that [^18^F]-FACBC PET underestimates the tumor spread in presence of non-enhancing tumor components, PET volumes could be used in these cases to indicate regions of likely high malignancy where margins should be maintained, potentially allowing for safer reduction in margins for the PET negative FLAIR volume where the tumor burden is likely lower.

The continued high rates of local treatment failure in glioblastomas have also led to interest in dose-escalation (DE) strategies for potentially improving local tumor control [42]. While studies using ce-T1 to define DE volume have shown limited results so far [43], a few recent studies using AA PET-guided DE show promising survival outcomes compared to conventional treatment [44–46], though there is currently no prospective randomized evidence to validate these findings. [^18^F]-FACBC PET has shown high sensitivity for detection of highly malignant tumor tissue and could be used in this context to identify tumor regions suitable for DE. In the current study, [^18^F]-FACBC -based PET volumes represented only a small fraction of the MR-defined CTVs, with the median PET GTV measuring approximately one-tenth the size of the median MR CTV. This could allow for increased doses to the metabolically active volume, possibly increasing efficacy of the treatment. Similarly, some studies have investigated the potential of increased linear energy transfer (LET) in particle RT dose planning, finding favorable possibilities of LET escalation without significantly increased doses to organs at risk [47, 48]. Proton RT is especially relevant for lower-grade gliomas with good prognoses, as the reduced doses to healthy brain tissue could minimize long-term effects on cognition, but could also have potential for DE in glioblastomas [3, 49]. Though results on the efficacy of proton RT in glioma are currently limited, accurate delineation of highly malignant tissue could be a possible future use of [^18^F]-FACBC PET in these cases.

## Limitations

There are several limitations in this study. Firstly, the cohort was heterogenous, including both low-and high-grade gliomas in both primary and recurrent settings to reflect clinical diversity. However, the impact of varying [^18^F]-FACBC uptake patterns between different types and grades were not considered in the statistical calculations, nor was the possible impact of the surgical cavity on MR GTVs for recurrent cases vs. primary cases. Sample sizes were also small, particularly for grade 2/3 astrocytomas and oligodendrogliomas, making it difficult to draw any firm conclusions about the impact of [^18^F]-FACBC in RT within these groups.

Furthermore, RT treatment volumes in this study were delineated based on pre-operative PET/MR images. While this represents clinical reality in some recurrent cases, and in primary cases where the tumor cannot be safely operated, RT of gliomas is typically performed after surgical resection with target delineation based on post-operative images. Future studies using post-operative PET/MR are needed to validate the generalizability of these findings.

In addition to the tracer-specific limitations of [¹⁸F]-FACBC discussed above, there are also general limitations of PET imaging compared to MR that could affect the accuracy of tumor delineation. Physically, PET has lower spatial resolution than MR which can impair delineation accuracy, especially for small or irregularly shaped lesions. Partial volume effects can also reduce sensitivity for small or infiltrative tumor foci [50], though [¹⁸F]-FACBC could have an advantage for detection of smaller lesions due to the higher TBR values compared to other AA tracers [51]. These limitations are important to consider when interpreting the observed discordance between PET and MR target volumes in this study. Since PET and MR provide inherently different types of information and are subject to different limitations, they are best used in a complementary manner for accurate and comprehensive target delineation.

Lastly, this study does not include correlation assessment of [^18^F]-FACBC uptake and recurrence sites. Further research is needed to evaluate how PET-based target delineation aligns with recurrence patterns, to explore the potential impact of [^18^F]-FACBC PET-guided RT on tumor control.

## Conclusions

[^18^F]-FACBC PET volumes were significantly smaller than MR based treatment volumes in gliomas with suspected non-enhancing tumor regions, while there was a high degree of concordance between ce-T1 only MR target volumes and [^18^F]-FACBC PET target volumes. [^18^F]-FACBC PET likely underestimates the true tumor extension in contrast negative tumors and tumors with non-enhancing components. However, [^18^F]-FACBC PET could be used in RT planning for glioma to identify regions of highly malignant tissue, which could facilitate margin reduction or DE strategies.

## Data Availability

The datasets generated and/or analyzed in the current study are not publicly available due to the European Union General Data Protection Regulations (GDPR) but are available from the corresponding author on reasonable request.

## Abbreviations

AA: Amino acid
[18F]-FACBC: Anti-1-amino-3-[18 F]-fluorocyclobutane-1-carboxylic acid
AC: Attenuation correction
BBB: Blood-brain barrier
CTV: Clinical target volume
ce-T1: Contrast-enhanced T1
DC: Dice similarity coefficient
DE: Dose escalation
EORTC: European Organisation for Research and Treatment of Cancer
FLAIR: Fluid-attenuated inversion-recovery
GTV: Gross tumor volume
HD: Hausdorff distance
IQR: Interquartile range
LET: Linear energy transfer
MR: Magnetic resonance
MPRAGE: Magnetization prepared rapid gradient echo imaging
OC: Overlap coefficient
PET: Positron emission tomography
RT: Radiotherapy
TBR: Tumor-to-background ratio
UTE: Ultrashort echo time
